# Effects of early continuous renal replacement therapy in critically ill patients requiring ECMO treatment: results of a randomised controlled trial

**DOI:** 10.1136/openhrt-2026-003963

**Published:** 2026-07-09

**Authors:** Xiaofang Wang, Ying Liu, Zhiyan Wang, Hong Wang, Chenglong Li, Jiakun Tian, Chi Wang, Xiaojun Liu, Jun Li, Yongjie Yin, Liwen Lv, Yang Wu, Binfei Li, Yiping Wang, Sheng Zhang, Xin Li, Craig S Anderson, Xin Du, Jianzeng Dong, Xiaotong Hou

**Affiliations:** 1 The First Affiliated Hospital, Zhengzhou University, Zhengzhou, China; 2 The Second Affiliated Hospital, Zhengzhou University, Zhengzhou, China; 3 Beijing Anzhen Hospital, Capital Medical University, Beijing, China; 4 The Second Hospital of Jilin University, Changchun, China; 5 Division of Cardiovascular Medicine, University of Nebraska Medical Center, Omaha, Nebraska, USA; 6 Guangxi Zhuang Autonomous Region People's Hospital, Nanning, Guangxi, China; 7 Chinese PLA General Hospital, Beijing, China; 8 Zhongshan City People’s Hospital, Zhongshan, China; 9 Sichuan Provincial People’s Hospital, Chengdu, China; 10 Taizhou Hospital of Zhejiang Province, Linhai, China; 11 Zhongshan Hospital Affiliated to Fudan University, Shanghai, China; 12 George Institute for Global Health, Faculty of Medicine, University of New South Wales, Sydney, New South Wales, Australia; 13 Institute for Science and Technology for Brain-inspired Intelligence, Fudan University, Shanghai, China; 14 Heart Health Research Center, Beijing, China

**Keywords:** Death, Sudden, Cardiac, Outcome Assessment, Health Care, Heart Arrest

## Abstract

**Background:**

Extracorporeal membrane oxygenation (ECMO) is increasingly used to treat patients with severe cardiac and pulmonary failure but the complications of acute kidney injury and fluid overload are common. We aimed to determine whether the early initiation of continuous renal replacement therapy (CRRT) can reduce 30-day mortality in patients with ECMO.

**Methods:**

The Evaluation of early CRRT intervention in patients with ECMO study was an investigator-initiated, multicentre, randomised, open clinical trial of patients undergoing ECMO who were randomly assigned to early initiation of CRRT (within 24 hours after ECMO) or conventionally indicated initiation of CRRT. All participants received standard diagnostic and therapeutic procedures. The primary endpoint was the 30-day mortality. A sample size of 500 patients was estimated to provide 80% power and a two-sided α of 0.05 to detect a 20% relative reduction in 30-day mortality assuming a rate of 70% in the usual care group. The primary analysis was unadjusted.

**Results:**

The study stopped early due to feasibility. Of the 131 patients assessed for eligibility, 40 were excluded based on the inclusion and exclusion criteria. Of the 91 randomised patients, 46 were assigned to the early CRRT group and 45 were assigned to the usual care group. In the intervention group, 35 patients (76.1%) received CRRT within 24 hours post-ECMO implementation vs 15 patients (33.3%) in the usual care group. In the intention-to-treat analysis, the primary outcome occurred in 20 patients (43.5%) in the early CRRT group and 21 patients (46.7%) in the usual care group (HR 0.93, 95% CI 0.50 to 1.72; p=0.75). There was no significant difference in serious adverse events between the two groups (11 (25.6%) vs 9 (21.4%); OR 1.26, 95% CI 0.46 to 3.45; p=0.65).

**Conclusions:**

Early implementation of CRRT in patients with ECMO showed no evidence of a difference in 30-day mortality compared with conventional initiation. However, the trial was substantially underpowered and clinically important benefits or harms cannot be excluded.

**Trial registration number:**

NCT03549923.

**WHAT IS ALREADY KNOWN ON THIS TOPIC**

- Acute kidney injury and fluid overload are common complications in patients receiving extracorporeal membrane oxygenation (ECMO), and continuous renal replacement therapy (CRRT) is frequently used to manage these issues.
- Previous observational studies have suggested that early initiation of CRRT may improve outcomes in ECMO patients, but robust randomised evidence has been lacking.

**WHAT THIS STUDY ADDS**

- The Evaluation of early CRRT intervention in patients with ECMO study found no significant difference in 30-day mortality between early initiation of CRRT within 24 hours post-ECMO over a conventional approach to the timing of CRRT.
- A substantial number of patients in the usual care group required CRRT later during hospitalisation.

**HOW THIS STUDY MIGHT AFFECT RESEARCH, PRACTICE OR POLICY**

- These findings suggest that a targeted strategy for CRRT, based on clinical indicators rather than routine immediate veno-arterial-ECMO (VA-ECMO) initiation, is effective and potentially optimises resource use in patients with VA-ECMO.

## Background

 Veno-arterial extracorporeal membrane oxygenation (VA-ECMO) is increasingly being used as a vital therapeutic option to provide short-term circulatory support for critically ill patients in cardiac shock.^1 2^ Despite improved survival rates, in-hospital mortality remains as high as 60% in those requiring VA-ECMO.^3^,^5^ The high in-hospital mortality stems from multiple factors: acute kidney injury (AKI), which occurs in 60% of cases and increases mortality,^6 7^ challenges in managing cannula flow and pressure, leading to fluid overload (FO) and the release of inflammatory cytokines which exacerbate organ dysfunction and worsen patient outcomes.^8 9^

Continuous renal replacement therapy (CRRT) aims to remove FO, manage AKI, remove inflammatory cytokines and stabilise haemodynamic parameters in patients on ECMO.^10^ Observational studies suggest that an early initiation of CRRT is associated with improved chances of recovery and survival on ECMO.^11^,^16^ According to data from Extracorporeal Life Support Organization (ELSO), CRRT utilisation increased from 18% to 40% over recent decades reflecting the growing enthusiasm among clinicians in applying CRRT.^16^ We undertook the evaluation of early CRRT intervention in patients with ECMO (ELITE) study to determine whether compared with standard use of CRRT, the initiation of CRRT within 24 hours of ECMO implementation will reduce 30-day mortality in patients requiring ECMO on top of routine standard of care.

## Methods

### Design

The ELITE study was an investigator-initiated and managed, multicentre, open-label, randomised controlled trial undertaken at 10 hospitals in China, as outlined in online supplemental table S1.^17^ Statistical analyses were performed by an independent statistician according to a statistical analysis plan published prior to database lock (https://osf.io/3a9kx/). The study was registered at ClinicalTrials.gov (NCT03549923). The Consolidated Standards of Reporting Trials (CONSORT) Statement checklist was used for the reporting of our study.^18^

### Inclusion and exclusion criteria

Patients were included if they were adults (age ≥18 years) who had VA-ECMO initiated within 24 hours for any cause. Patients were excluded if they had any of the following: severe abnormalities of biochemistry including a blood urea nitrogen level >112 mg/dL, serum potassium >6.5 mmol/L or pH<7.2; urine volume <200 mL over 12 hours; unresponsive pulmonary oedema to diuretic treatment; requirement for oxygen >5 L/min to maintain an oxygen saturation >95% or assisted breathing with an inspired oxygen fraction (FiO2)>50%; or a history of chronic kidney disease with estimated glomerular filtration rate <30 mL/min (online supplemental table S2).

### Procedures

All eligible patients were centrally randomised (1:1) via a smartphone application and secure website to either early CRRT (within 24 hours after implementation of VA-ECMO regardless of indication for CRRT) or usual care (no CRRT unless there was a conventional indication). Randomisation was stratified by age (<65 vs ≥65 years) and use of ECMO for extracorporeal cardiopulmonary resuscitation (extracorporeal cardiopulmonary resuscitation (ECPR), yes vs no). Patients allocated to early CRRT had CRRT initiated as soon as possible after randomisation and the treatment was to be maintained for ≥12 hours. The decision to initiate CRRT in the usual care group was at the discretion of the attending physician.

### Outcomes

The primary endpoint was all-cause mortality at 30 days post-randomisation. Key secondary outcomes were success in weaning from ECMO (defined as survival >24 hours after cessation of ECMO), overall cost of hospitalisation, and cardiac versus non-cardiac cause-specific mortality at 30 days.

All serious adverse events (SAEs) that occurred to the end of follow-up were also collected according to standard definitions. Adverse events of special interest were major bleeding, severe cardiac arrhythmias, ventilator-associated pneumonia, bloodstream infections, surgical-associated infection, limb ischaemia of any cause and stroke.

### Statistical analysis

A total of 248 patients in each arm was estimated to provide 80% power and a 2-sided α 0.05 to detect a 20% relative risk reduction in 30-day mortality from a rate of 70% in the usual care group. Analyses were performed according to the intention-to-treat principle. A per-protocol analysis was also undertaken in those who adhered to the treatment assignments. Baseline characteristics were presented as frequencies or percentages. Continuous data were presented as the mean (±SD) for data that followed a normal distribution, and as medians (IQR) for data that followed non-normal distribution. Comparisons of the baseline characteristics were conducted by Pearson χ² or Fisher’s exact tests for continuous variables or using students’ t or Mann-Whitney tests for categorical variables. The Kaplan-Meier method and log-rank test were used to compare the time from randomisation to the occurrence of death. Cox proportional hazard models and logistic regression models were used to determine HRs and ORs, respectively, with associated 95% CI for primary and secondary outcomes. Subgroup analysis was conducted according to age (<65 vs ≥65 years) and ECMO indication (ECPR or not). All analyses were conducted with SAS (V.9.4) and R (V.4.3.0). A p<0.05 was set as statistically significant.

## Results

### Patients

**Figure 1:**
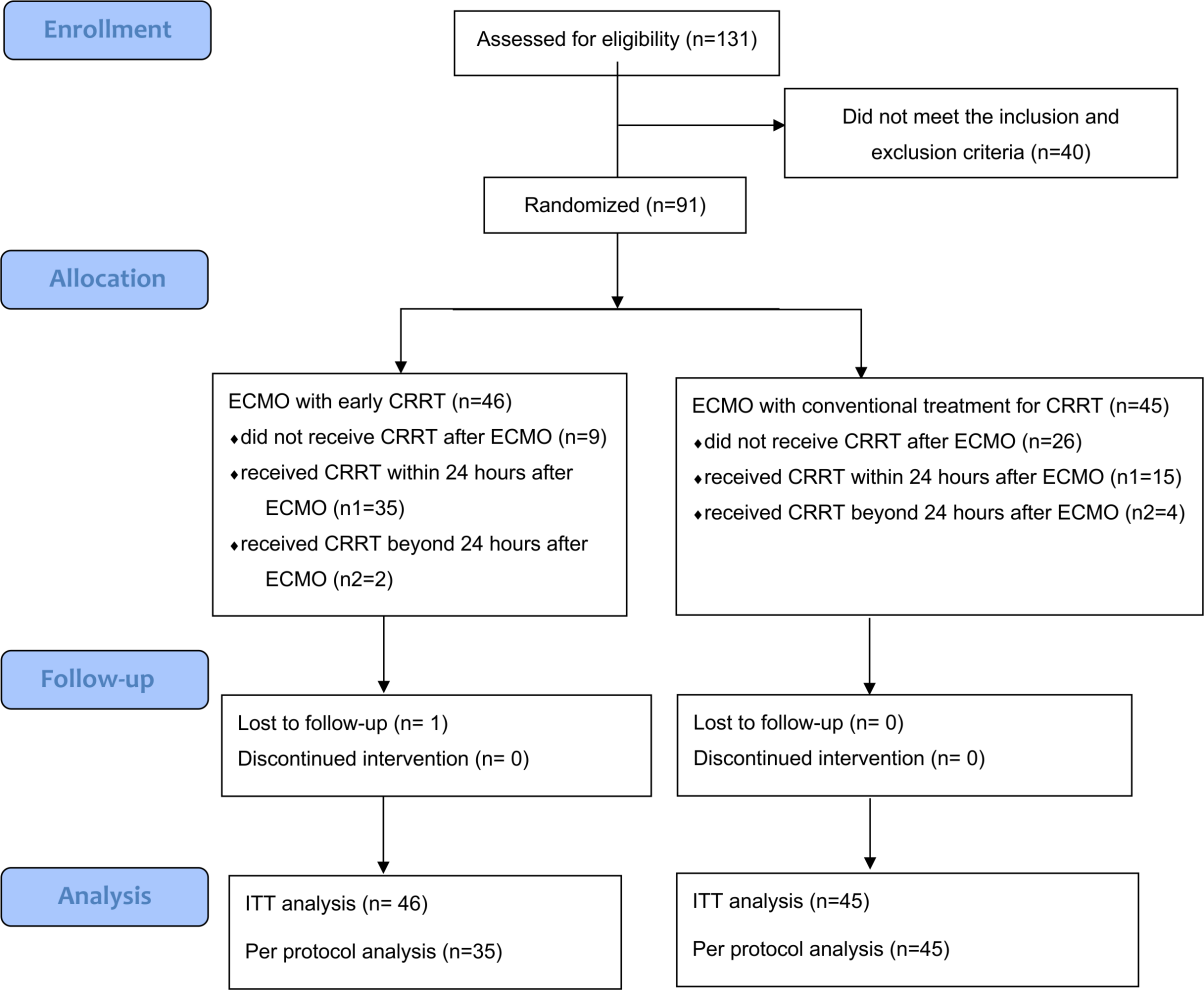
Patient flow chart. CRRT, continuous renal replacement therapy; ECMO extracorporeal membrane oxygenation, ITT, intention-to-treat.

The trial was stopped early due to poor recruitment and lack of continued funding. Overall, 131 patients were assessed for eligibility and 91 patients were randomised between 18 December 2018 and 22 October 2021 (figure 1). There were 46 participants in the early CRRT group but 9 did not receive this treatment and 2 received CRRT after 24 hours of ECMO insertion. Of the 45 participants in the usual care group, 15 and 4 participants received CRRT within and after 24 hours of ECMO insertion, respectively. One participant in the early CRRT group was lost to follow-up.

Table 1 presents the baseline characteristics of the participants. The average age was 53.4 years, and 31.9% were female. At the time of randomisation, their mean systolic blood pressure (SBP) was 90.1 mm Hg, and the mean arterial pressure (MAP) was 70.4 mm Hg. The median arterial lactate level was 5.1 mmol/L (2.3 to 7.9 mmol/L), and the median creatinine level was 125.5 µmol/L (83.0 to 152.5 µmol/L). The mean Glasgow Coma Scale score was 7.5 (5.0 to 15.0).

**Table 1:** Baseline characteristics of participants

	Overall (n=91)	Early CRRT (n=46)	Usual care (n=45)
Age	53.4 (13.4)	54.1 (13.3)	52.7 (13.5)
Female	31 (31.9)	16 (34.8)	15 (33.3)
BMI	24.0 (4.0)	23.9 (4.1)	24.1 (3.8)
Medical history			
Hypertension	38 (42.2)	21 (47.7)	17 (37.8)
Diabetes mellitus	19 (21.1)	7 (15.9)	12 (26.7)
Heart failure	10 (11.1)	5 (11.4)	5 (11.1)
Chronic kidney disease	1 (1.1)	0 (0)	1 (2.2)
Stroke	6 (6.7)	2 (4.6)	4 (8.9)
Myocardial infarction	10 (11.1)	4 (9.0)	6 (13.3)
PCI	9 (10.0)	4 (9.0)	5 (11.1)
Clinical parameters			
Lactate	5.1 (2.3, 7.9)	4.8 (2.1, 6.7)	5.4 (2.5, 9.0)
SBP	90.1 (19.2)	89.2 (17.3)	91.0 (20.9)
DBP	60.7 (15.9)	60.6 (14.5)	60.8 (17.3)
MAP	70.4 (15.8)	70.1 (14.3)	70.8 (17.3)
Creatinine	125.5 (83.0, 152.5)	130.4 (88.0, 150.0)	120.5 (78.0, 155.0)
eGFR	80.7 (64.8, 93.7)	81.5 (62.9, 86.6)	80.0 (68.8, 98.9)
GCS score	7.5 (5.0, 15.0)	8.0 (5.0, 15.0)	7.0 (5.0, 15.0)
APS score	19.5 (16.0, 23.5)	19.0 (15.0, 23.0)	20.0 (17.0, 24.0)
SOFA score	7.0 (5.0, 9.0)	7.0 (5.0, 9.0)	7.00 (5.0, 9.0)
APACHEII score	19.5 (16.0, 23.5)	19.0 (15.00, 23.00)	20.00 (17.00, 24.00)
Treatment at randomisation			
Mechanical ventilation (%)	74 (81.3)	37 (80.4)	37 (82.2)
Vasoactive drugs (%)			
Norepinephrine	54 (59.3)	24 (52.5)	30 (66.7)
Epinephrine	16 (17.6)	10 (21.7)	6 (13.3)
Dobutamine	21 (23.1)	12 (26.1)	9 (20)
Vasopressin	0 (0)	0 (0)	0 (0)
Milrinone	2 (2.2)	0 (0)	2 (4.4)
Implementation of ECMO for ECPR	26 (28.9)	11 (23.9)	15 (33.3)

Data are n (%), mean (SD) or median (IQR).

APACHEII, Acute Physiologic Assessment and Chronic Health Evaluation; APS, Acute Physiology Score; BMI, body mass index; CRRT, continuous renal replacement therapy; DBP, diastolic blood pressure; ECPR, Extracorporeal Cardiopulmonary Resuscitation; eGFR, estimated glomerular filtration rate; GCS, Glasgow Coma Scale; HF, heart failure; MAP, mean arterial pressure; PCI, percutaneous coronary intervention; SBP, systolic blood pressure; SOFA, Sequential Organ Failure Assessment.

At the time of randomisation, over 80% of patients were on mechanical ventilation, and approximately 60% were receiving treatment with vasoactive drugs. A total of 26 patients (28.9%) underwent ECMO due to ECPR.

**Figure 2:**
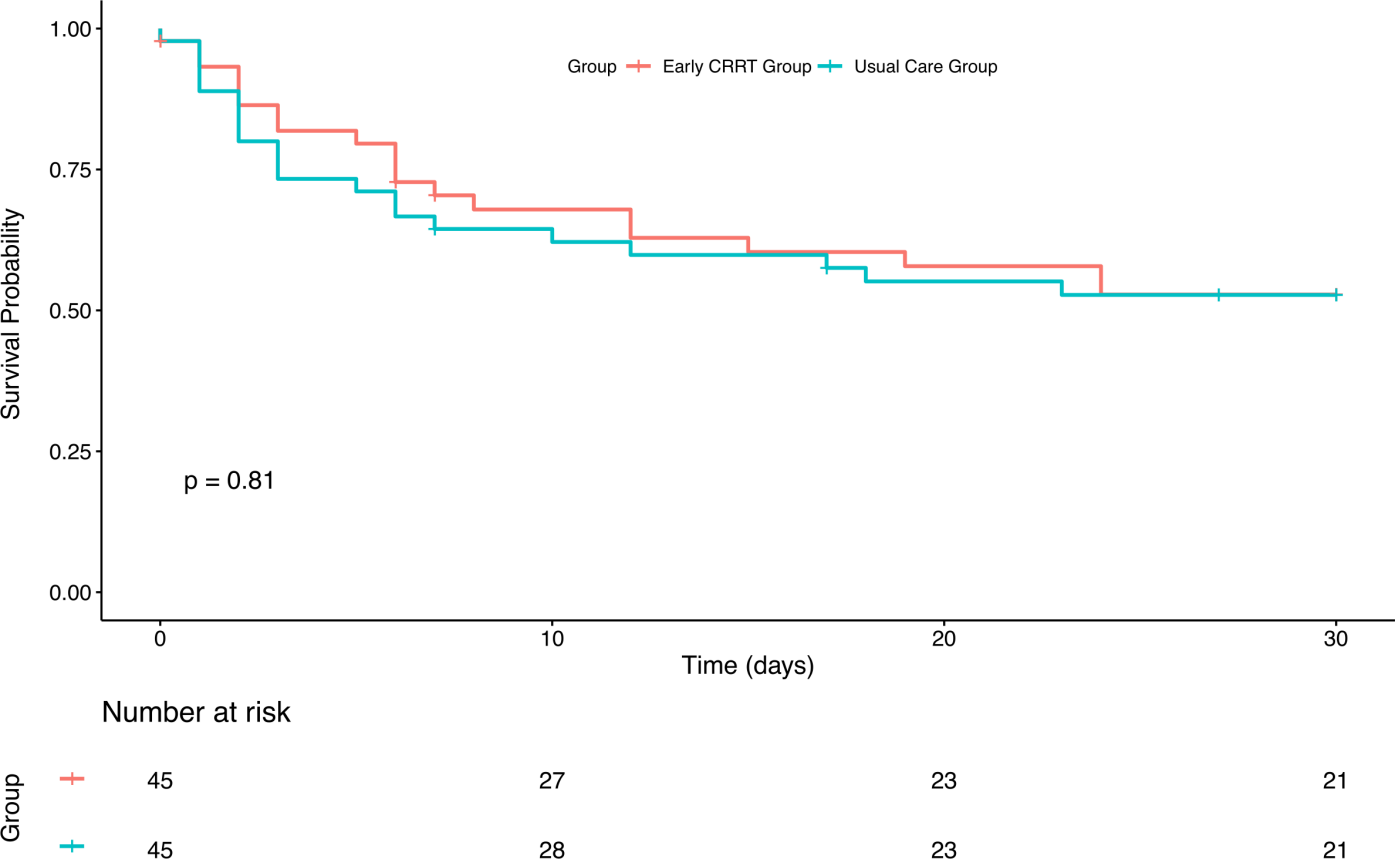
Survival curves of the primary outcome. CRRT, continuous renal replacement therapy.

There was no significant difference in 30-day mortality between the groups, which occurred in 20 (43.5%) participants in the early CRRT group and 21 (46.7%) of participants in the usual care group (HR 0.93, 95% CI 0.50 to 1.72; p=0.82) (table 2 and figure 2). In per-protocol analysis, the trend was for 30-day mortality to be higher with early CRRT compared with usual care (54.3% vs 46.7%; HR 1.12, 95% CI 0.60 to 2.08; p=0.50), as shown in online supplemental table S3 and figure S1.

**Table 2:** Primary and secondary outcomes and safety

Outcome	Early CRRT	Usual care	HR/OR (95% CI)	P value
Primary				
30-day death	20 (43.5)	21 (46.7)	0.93 (0.50 to 1.72)	0.75
Secondary				
Success in weaning from ECMO	32 (72.7)	27 (60)	0.56 (0.23 to 1.37)	0.2
In-hospital cost USD	35 826 (24 551, 52 870)	31 884 (21 044, 46 928)	–	0.41
Cardiac death at 30 days	10 (21.7)	7 (15.6)	1.37 (0.52 to 3.62)	0.45
Non-cardiac death at 30 days	10 (21.7)	14 (31.1)	0.70 (0.31 to 1.59)	0.31
Safety				
Adverse events	11 (25.6)	9 (21.4)	1.26 (0.46 to 3.45)	0.65

Adverse events include bleeding, severe arrhythmia, ventilator-associated pneumonia, bloodstream infection, procedure-associated infection, limb ischaemia at any cause and stroke.

*Defined as being alive >24 hours after ECMO withdrawal.

†An OR less than 1 indicates that higher success rate in weaning from ECMO in the early CRRT group than the control group.

CRRT, continuous renal replacement therapy; ECMO, extracorporeal membrane oxygenation.

There were no significant differences in secondary outcomes between the groups (table 2). The median in-hospital cost was US$35 826 for the early CRRT group and US$31 884 for the usual care group. There were 11 adverse events (25.6%) in the early CRRT and 9 adverse events (21.4%) in the usual care group. Further details are outlined in online supplemental table S4.

**Figure 3:**
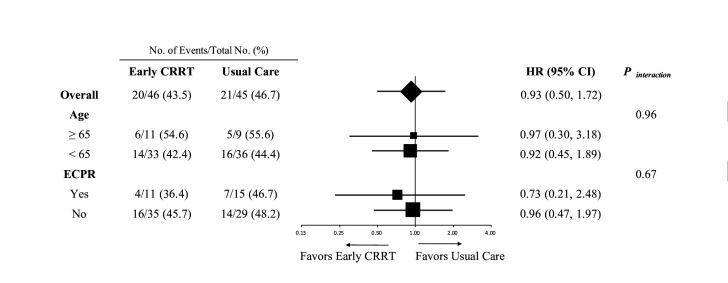
Subgroup analysis of the primary outcome. CRRT, continuous renal replacement therapy; ECPR, extracorporeal cardiopulmonary resuscitation.

The neutral effect of early CRRT on 30-day mortality was consistent across predefined age groups and indications for ECMO (figure 3 and online supplemental figure S2).

## Discussion

To the best of our knowledge, this is the first clinical trial to assess whether early CRRT improves survival in critically ill patients requiring ECMO. We found no difference in 30-day all-cause mortality between early CRRT treatment and usual care when CRRT was used according to conventional indications. Additionally, there were no differences between the groups in the success of weaning from ECMO, in-hospital costs, cause-specific mortality or adverse events. Given that only 91 patients were enrolled, substantially fewer than the planned 500, the study had limited statistical power and wide CIs. Therefore, these results should be interpreted with caution.

The critical nature and requirement for intensive management posed challenges to the recruitment of patients into our study. Consequently, the neutral findings may be attributed to low statistical power to detect potentially meaningful but subtle differential effects. Furthermore, crossover in this study may also have contributed to the results. In the intervention group, 24% of patients did not receive CRRT treatment within 24 hours post-randomisation, while in the usual care group, one-third of patients received CRRT treatment within 24 hours post-randomisation. This treatment crossover reduced the separation between groups and may have diluted the treatment effect. Per-protocol findings should therefore be interpreted with caution, as they are susceptible to selection bias and loss of randomisation, and should be considered exploratory. Additionally, the heterogeneity in participants and varied reasons for ECMO implementation suggest that mortality might have been influenced more by the severity of the primary disease rather than by the timing of CRRT.

Several studies have investigated the timing of CRRT initiation in patients with AKI, yielding controversial results. A meta-analysis of randomised trials showed a survival benefit of early CRRT in AKI patients.^19^ In the early versus late initiation of renal replacement therapy in critically ill patients with AKI (ELAIN) study involving 231 critically ill patients with AKI,^20^ early CRRT within 8 hours of diagnosing the Kidney Disease: Improving Global Outcomes (KDIGO) stage 2, there was reduced 90-day mortality compared with delayed CRRT (initiated within 12 hours of KDIGO stage 2 diagnosis or not initiated at all). However, the Artificial Kidney Initiation in Kidney Injury study, which included 620 patients with KDIGO stage 3 AKI, found no difference in 60-day mortality between the early and delayed CRRT groups.^21^ A meta-analysis of nine randomised trials revealed that ‘early’ initiation of CRRT is not associated with a reduced mortality in critically ill patients. ^22^In ECMO patients, no randomised trials have been conducted to evaluate the optimal timing for CRRT initiation but a meta-analysis of observational studies showed a potential benefit with an early initiation.^16^ However, these findings are limited by the nature of observational studies.

CRRT is commonly used in ECMO patients, with over 50% receiving combination therapy.^23^ Our study reflects real-world VA-ECMO treatment for cardiac shock, with CRRT usage in the usual care group aligned with observational cohorts.^23^ Data from the ELSO cohort indicates an in-hospital mortality rate of approximately 55% in ECMO patients. Our 30-day mortality rate of 45% closely aligns with this figure, suggesting the relevance of our study’s conclusions^24 25^ (online supplemental table S5). Early CRRT administration in ECMO patients failed to improve outcomes but also showed no harmful effects. Our study found comparable rates of SAEs including bleeding, stroke and bloodstream infection between the early CRRT and usual care groups. Additionally, the overall cost of ECMO was not significantly increased by adding CRRT.

We acknowledge our study has several limitations. First, the complexity inherent in patients with ECMO posed significant challenges in conducting randomised controlled trials where a sufficiently large and representative sample is required. Despite our best efforts to enrol as many patients as possible, our study did not reach the target number of patient as planned. The absolute difference in mortality between groups was small; however, given the limited sample size, the study lacked the ability to detect moderate but clinically important differences. Importantly, the CI around the HR includes the possibility of both meaningful benefit and harm, indicating that clinically relevant effects cannot be excluded. This limitation is further exacerbated by the global COVID-19 pandemic, which not only impacted on the availability of eligible patients but also introduced additional constraints on our ability to conduct the research. Our study adopted broad inclusion criteria to capture the diverse range of patients undergoing ECMO treatment but this approach introduced heterogeneity in the clinical characteristics of participants. This variability may influence the interpretation of our results, as the effectiveness of early CRRT strategies may vary significantly across different subgroups of patients. Finally, as our study was open, this may have influenced the therapeutic decisions of attending physicians and introduced confounding. A major challenge in interpreting the results is the substantial treatment crossover between groups. Approximately 24% of patients in the early CRRT group did not receive early treatment, while about one-third of patients in the usual care group received early CRRT. This degree of contamination effectively reduced the contrast between treatment strategies and biases the results toward the null, making it difficult to detect true differences. As a result, the study may better reflect a comparison of treatment preferences rather than a strict early versus delayed strategy.

In conclusion, early CRRT initiation in patients receiving ECMO showed no evidence of a difference in mortality compared with a conventional strategy. However, due to substantial underpowering and treatment crossover, the findings are inconclusive, and clinically meaningful benefits or harms cannot be excluded. Larger, adequately powered trials are required.

### Trial management

The Coordination Centre, Steering Committee, International Advisory Committee and Data and Safety Monitoring Board of the ELITE trial are listed in online supplemental table S6.

## Supplementary material

10.1136/openhrt-2026-003963 online supplemental file 1

## Data Availability

Data are available on reasonable request.
