# Supplementary material for: Effects of early continuous renal replacement therapy in critically ill patients requiring ECMO treatment: results of a randomised controlled trial
Source: Open Heart. 2026 Jul 9;13(2):e003963. doi: 10.1136/openhrt-2026-003963 (PMC13358276; doi:10.1136/openhrt-2026-003963)
Supplement: online supplemental file 1 [file openhrt-13-2-s001.docx]

**Effects of early continuous renal replacement therapy in critical ill patients requiring ECMO treatment: results of a randomized controlled trial**

**Supplement materials**

**Table S1 ELITE study centers and investigators**

| **Centers** | **Investigators** | **Location** |
| --- | --- | --- |
| The Secondary Affiliated Hospital of Zhengzhou University | Xiaojun Liu | Zhengzhou, Henan province |
| The First Affiliated Hospital of Zhengzhou University | Jun Li | Zhengzhou, Henan province |
| The Second Hospital of Jilin University | Yongjie Yin | Changchun, Jilin province |
| The People’s Hospital of Guangxi Zhuang Autonomous Region | Liwen Lv | Nanning, Guangxi Zhuang Autonomous Region |
| Chinese PLA General Hospital | Yang Wu | Beijing |
| Beijing Anzhen Hospital | Xiaotong Hou | Beijing |
| Zhongshan City People’s Hospital | Binfei Li | Zhongshan, Guangdong province |
| Sichuan Provincial People’s Hospital | Yiping Wang | Chengdu, Sichuan province |
| Taizhou Hospital of Zhejiang Province | Sheng Zhang | Taizhou, Zhejiang province |
| Zhongshan Hospital Affiliated to Fudan University | Xin Li | Shanghai |

**Table S2 Inclusion and exclusion criteria for ELITE study**

| Inclusion criteria |
| --- |
| - Patients receiving VA-ECMO for any reason within 24 hours |
| - Provision of informed consent |
|  |
| Exclusion criteria |
| - Age < 18 years - Receiving ECMO bridging to heart transplantation |
| - With convention indication of CRRT: AKI prior to enrollment caused by any reason, at least one of the following criteria is met: |
| 1. Severe hyperkalemia (> 6.5 mmol/L) |
| 1. Metabolic acidosis (pH < 7.2) |
| 1. Pulmonary edema |
| 1. Blood urea nitrogen level > 112 mg/dL |
| 1. Oliguria (urine output < 200 mL/12h) for more than 72 hours |
| - CKD, with estimated GFR<30 mL/min |
| - Have already initiated CRRT - Active hemorrhage/thrombotic thrombocytopenic purpura - Receiving ECMO again during hospitalization or respiratory failure has already initiated VV-ECMO or extracorporeal carbon dioxide removal device before the initiation of VA-ECMO of this time |

AKI: chronic kidney disease; CKD: chronic kidney disease; CRRT: continuous renal replacement therapy; ECMO: extracorporeal membrane oxygenation; GFR: glomerular filtration rate; VA-ECMO: veno-arterial extracorporeal membrane oxygenation; VV-ECMO: veno-venous extracorporeal membrane oxygenation.

**Table S3 Primary outcome and secondary outcomes by per-protocol analysis**

| Outcome | Per protocol analysis | | HR/OR (95%CI) | P value |
| --- | --- | --- | --- | --- |
|  | Early CRRT | Conventional treatment |  |  |
| **Primary outcome** |  |  |  |  |
| 30-day death, n (%) | 19 (54.3) | 21(46.7) | 1.12 (0.60,2.08) | 0.5 |
| **Secondary outcomes** |  |  |  |  |
| Success in weaning from ECMO | 23 (67.7) | 27 (60) | 0.72 (0.28, 1.83) * | 0.49 |
| In hospital cost (US dollar), Median(IQR) | 35826.1 (23188.4, 56913.04) | 31884.1(21043.5,46927.5) | - | 0.36 |
| Cardiac death at 30 days | 10 (28.6) | 7 (15.6) | 1.74 (0.66, 4.57) | 0.16 |
| non-cardiac death at 30 days | 9 (25.7) | 14 (31.1) | 0.80 (0.35, 1.85) | 0.6 |
| **Safety** |  |  |  |  |
| **Adverse events** | 9 (25.7) | 9 (21.4) | 1.27 (0.44, 3.65) | 0.66 |

- Success in weaning from ECMO was defined as 24 hours alive after ECMO withdrawal
- Adverse events include bleeding, severe arrythmia, ventilator-associated pneumonia, bloodstream infection, procedure-associated infection, limb ischemia at any cause and stroke
- Odds ratios and 95%CI were represented in success in weaning from ECMO and adverse events.

* An OR less than 1 indicates that higher success rate in weaning from ECMO in the early CRRT group than the control group.

**Table S4 Adverse events by ITT and per-protocol analysis**

|  | ITT analysis | | **P value** | Per protocol analysis | | **P value** |
| --- | --- | --- | --- | --- | --- | --- |
| SAE | Early CRRT | Conventional treatment |  | Early CRRT | Conventional treatment |  |
| Bleeding | 5 (11.6) | 4 (9.5) | 0.26 | 4 (11.4) | 4 (9.5) | 0.28 |
| Severe arrythmia | 1 (2.3) | 1 (2.4) | 0.51 | 1 (2.9) | 1 (2.4) | 0.50 |
| Ventilator-associated pneumonia | 2 (4.7) | 1 (2.4) | 0.38 | 0 (0) | 1 (2.4) | 0.55 |
| Bloodstream infection | 3 (7.0) | 1 (2.4) | 0.26 | 3 (8.6) | 1 (2.4) | 0.20 |
| Procedure-associated infection | 1 (2.3) | 1 (2.4) | 0.51 | 1 (2.9) | 1 (2.4) | 0.50 |
| Limb ischemia at any cause | 1 (2.3) | 1 (2.4) | 0.51 | 1 (2.9) | 1 (2.4) | 0.50 |
| Stroke | 4 (9.3) | 4 (9.5) | 0.29 | 3 (8.6) | 4 (9.5) | 0.30 |
| Other | 3 (7.0%) | 0 (0%) | 0.13 | 3 (7.0%) | 0 (0%) | 0.09 |

**Table S5 Comparison of patients’ characteristics in ELITE and ELSO registry**

|  | ELITE | ELSO^1^ | ELSO^2^ |
| --- | --- | --- | --- |
| Number of patients | 91 | 2699 | 15172 |
| Mortality | 45% 30-day death | 58.6% in hospital death | 53.9% in hospital death |
| Recruitment period | 2018-2021 | 2002-2012 | 2017-2019 |
| Age (mean (SD)) | 55.0 (42.0, 63.0) | 54.0 (42.0, 63.0) | Not available |
| Female (%) | 31 (34.1) | 862 (31.9) | 4967 (32.7) |
| Weight (mean (SD) | 67.0(60.0,79.0) | 75.0 (64.0, 88.9) | Not available |
| Days of ECMO | 3.75 (2.42, 5.46) | 4.00 (1.96, 6.83) | Not available |
| pH (median (IQR)) | 7.37 (7.3,7.44) | 7.31 (7.21, 7.40) | Not available |
| MAP | 72.7(56.7, 83.3) | 59 (49, 70) | Not available |

1. Smith M, Vukomanovic A, Brodie D, Thiagarajan R, Rycus P, Buscher H. Duration of veno-arterial extracorporeal life support (VA ECMO) and outcome: an analysis of the Extracorporeal Life Support Organization (ELSO) registry. Crit Care. 2017;21(1):45. doi:10.1186/s13054-017-1633-1

2. Fernando SM, MacLaren G, Barbaro RP, et al. Age and associated outcomes among patients receiving venoarterial extracorporeal membrane oxygenation-analysis of the Extracorporeal Life Support Organization registry. Intensive Care Med. 2023;49(12):1456-1466. doi:10.1007/s00134-023-07199-1

**Table S6 Trial management**

| **Trial Coordination Center:** | Heart Health Research Center (HHRC), Beijing, China |
| --- | --- |
| **Steering Committee:** | Jianzeng Dong, Xiaotong Hou, Craig Anderson, Xin Du, Hong Wang, Simon Finfer |
| **International Advisory Committee:** | Anushka Patel, Kazem Rahimi, Daniel Brodie, Roberto Lorusso, Alain Combes |
| **Data and Safety Monitoring Board:** | Bruce Neal, Jicheng Lv, Xiang Guo |

**Figure S1 Survival curves of the primary outcome by pre-protocol analysis**

Abbreviation：CRRT: Continuous renal replacement therapy

**Figure S2 Subgroup analysis of the primary outcome by per-protocol analysis**


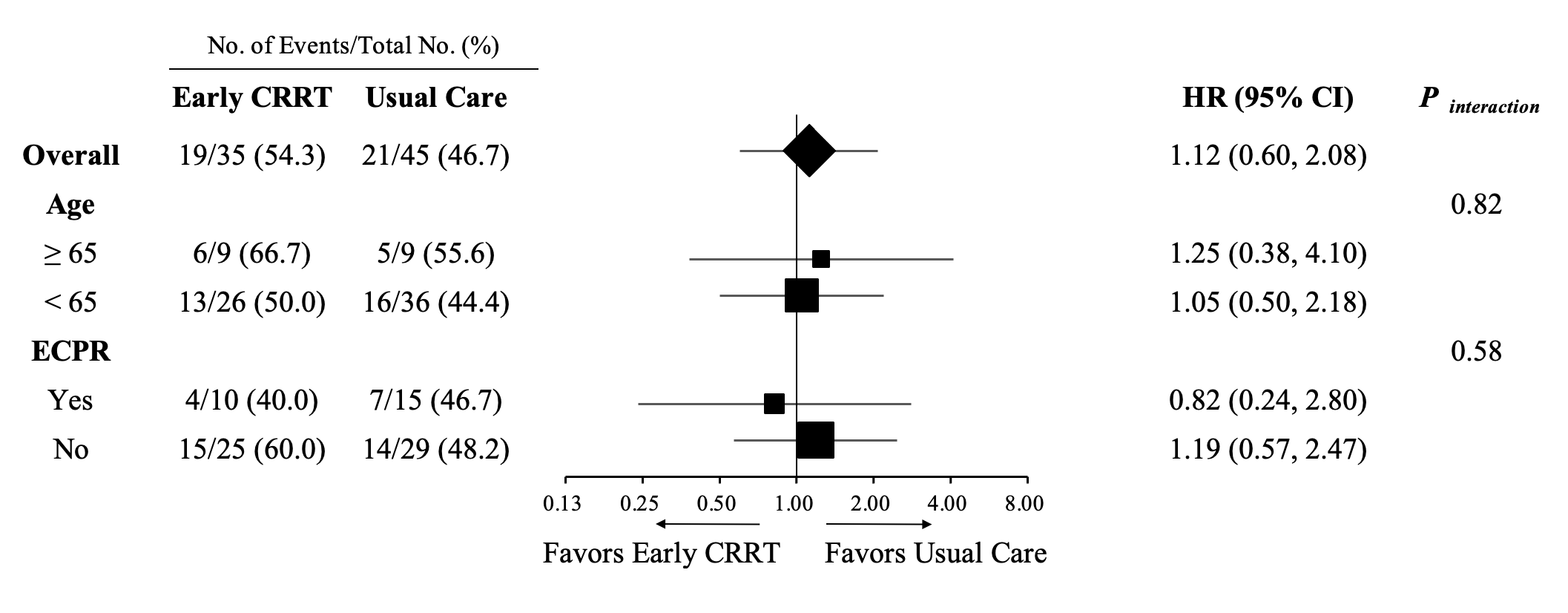


Abbreviation：

HR: Hazard ratio; 95%CI: 95% Confidence interval; ECPR: Extracorporeal cardiopulmonary resuscitation; CRRT: Continuous renal replacement therapy
